# A hitchhikers’ guide to the terminology of accreditation processes for health professionals and institutions

**DOI:** 10.12688/mep.19566.1

**Published:** 2023-02-27

**Authors:** Nuno Sousa, Andre Santa-Cruz, Antonio Melo, Camila Sousa, Fernanda Marques, Hugo Leite-Almeida, Lucimara Souza, Marco Marangoni, Marcia Raia, Maurilio Santos, Nuno Goncalves, Pedro Morgado, Pedro Souza, Rita Matos Sousa, Sara Gomes, Wilfredo Santos, Beatriz Araújo, Eliana Amaral, Vitor Pereira, Peter Scoles, MAPS

**Affiliations:** 1 School of Medicine, University of Minho, Portugal; Clinical Academic Center (2CA), Braga, Portugal; 2 Inspirali Education, Minas Gerais, Brazil; 3 Santa Casa de Misericórdia de Belo Horizonte, Minas Gerais, Brazil; 4 Medical School, Integrado University Center, Campo Mourao, Brazil; 5 School of Medicine, UniEduk group, UniMax Campus, Indaiatuba, Brazil; 6 School of Medical Sciences, University of Campinas, Campinas, Brazil; 7 Sidney Kimmel Medical College, Thomas Jefferson University, Philadelphia, USA

**Keywords:** accreditation, certification, licensing, assessment, evaluation, outputs, outcomes, quality

## Abstract

Accreditation processes for health care professions are designed to ensure that individuals and programs in these fields meet established standards of quality and effectiveness. The accelerating pace of globalization in the health care professions has increased the need for a shared understanding of the vocabulary of evaluation, assessment, and accreditation. The psychometric principles of valid and reliable assessment are commonly accepted, but the terminology is confusing. We believe that all stakeholders – evaluators, faculty, students but also the community – will benefit from a shared language and common set of definitions. We recognize that not all readers will agree with the definitions we propose, but we hope that this guide will help to ensure clarity, consistency, transparency, and fairness, and that it will promote through the stimulation of a debate greater collaboration across national and international boundaries.

## Introduction

It is generally accepted that that reliable accreditation improves health care education and assures comparable standards for students, educators, patients, health care providers, institutions, and the public ( Bandiera *et al.,* 2020; Stalmeijer *et al.,* 2022). The use of these standards implies in different dimensions the measurement of distinct parameters, followed by their analysis, which often implies a judgement; as a result, the topic of evaluation and assessment are critical for accreditation processes. Ultimately, these processes will assure quality and even more important a culture of quality improvement. In fact, continuous quality improvement is a cornerstone of effective delivery of health care ( Bendermacher *et al.,* 2021). The culture of continuous quality improvement is grounded in principles that also guide accreditation processes ( Amaral & Norcini, 2023; Frank *et al.,* 2020), including:

1. When performance is measured, performance improves.
2. Patients, families, and communities are the ultimate consumers of health care delivery and health care education. Health care systems and health care professionals are ultimately accountable to the public.
3. The public relies on health care professionals to self-govern and self-regulate their own practice. Professional autonomy coexists with public accountability.
4. Continuous quality improvement in health care services includes continuous quality improvement in assessment and accreditation practices.
5. Stakeholders in accreditation programs include patients, health care professionals, employers, and government agencies charged with protecting the health and welfare of all citizens.
***6.*** Resulting credibility is essential to the success in the implementation of communal health policies both at small/local and large/global scales.

## The terminology commonly used in accreditation processes

Common understanding and consistent use of words and phrases is essential when applying standards within systems and comparing standards across systems. The terminology of assessment and accreditation is commonly and frequently confused by experts and novices alike. Many of the terms have more than one meaning, depending on the context in which they are used. Confusion increases when the terms are translated out of context and across more languages and the distinctions are often not trivial.

We propose the following lexicon as basis for stimulating a debate around the terms, and ultimately the topics, that are relevant for accreditation processes (see Table 1).

**Table 1.** Lexicon of some of the common terms (listed in alphabetic order) used in accreditation processes.

• **Accreditation:** The process of formally recognizing an institution, program, or service as meeting certain standards of quality and effectiveness.
• **Assessment:** The process of observation and/or examination to measure specific attributes of students (e.g., knowledge, skills, and professionalism), professionals and/or institutions.
• **Benchmarking**: Comparison of an institution, program, or service to other similar institutions, programs, or services to identify best practices and areas for improvement.
• **Certification**: The process of providing formal recognition that an individual or organization has demonstrated a specific set of skills or knowledge in a particular field or area of expertise.
• **Continuous quality improvement**: The ongoing process of evaluating and improving the quality and effectiveness of an institution, program, or service
• **Evaluation**: The process of interpreting the results of assessment to inform judgments about an individual, program, or institution.
• **Entrustable professional activity**: A procedure or process generally considered to be required for patient care that an individual can be trusted to perform in a given health care context, once sufficient competence has been demonstrated.
• **Licensure**: The process of obtaining a license to legally practice within a jurisdiction
• **Outcomes assessment:** The process of gathering information regarding the effectiveness of an institution, program, or service in achieving its stated goals and objectives.
• **Peer review:** A process in which experts in within a particular discipline evaluate the quality and effectiveness of an individual, institution, program, or service.
• **Quality assurance:** The process of evaluating and ensuring that an institution, program, or service meets established standards.
• **Rubrics:** Tools used to evaluate the performance of an institution, program, or service against established standards. They typically outline specific criteria and provide a scoring system for evaluating performance.
• **Self-regulation:** A process in which an institution, program, or service evaluates its own performance against established standards.
• **Site visit**: A process in which a team of experts visits an institution, program, or service to evaluate its performance and compliance with established standards.
• **Stakeholders:** Individuals or groups who have a vested interest in the quality and effectiveness of an institution, program, or service, such as students, faculty, employers, and the public.
• **Standards:** The criteria that institutions, programs, or services must meet in order to be accredited or deemed effective.

In the paragraphs that follow, we will explore the relationships among these terms as they are commonly and frequently confused by experts and novices alike – this will be presented as practical tips to provide guidance in these processes. Many of the terms have more than one meaning, depending on the context in which they are used and confusion increases when the terms are translated out of context across one or more languages. Obviously, it is not the intention of this manuscript to impose these terms and their relationship, but rather to provide some guidance and to stimulate a debate around the accreditation processes.

### Tip 1 - Accreditation, certification, and licensing

The processes of accreditation, certification and licensure ensure that individuals or organizations meet certain standards or requirements in their field ( Bandiera *et al.,* 2020). While these concepts are often used interchangeably, they refer to different things.

Accreditation is the formal recognition that an institution or program meets certain standards of quality. Accreditation is often required for government funding or recognition from other organizations. Accreditation assures colleges and universities that the credentials of its graduates will be accepted by employers and government agencies. Depending on the purpose of accreditation, accrediting bodies may be governmental agencies, independent non-governmental organizations acting on behalf of the government, or independent groups acting on behalf of a professional society. Accrediting agencies typically provide an institution a set of standards to guide a self-study process, and then evaluate compliance with these standards. Accreditation by governmental agencies or organizations acting on behalf of a government is usually required for health care colleges and health care systems.

In the health care professions, certification is the process of attesting that an individual has met the generally recognized criteria for practice within a professional discipline. This may involve completing a course of study, passing an exam, and gaining a certain amount of experience. Certification is often provided by professional organizations or industry groups. Although certification may not be required for licensure, employers may require certification as a condition for practice.

Most educators and regulatory agencies recognize that certification at start of a career does not assure continued competency. Re-certification is now required in many health professions and usually includes documentation of commitment to life-long learning, completion of required continuing education courses, and satisfactory completion of a knowledge examination. 

Licensure is the process of granting permission to an individual or organization to engage in a certain activity or practice. This is typically done by a government agency. Licensing in aviation, engineering, and health care is designed to protect the public by ensuring that individuals and organizations have the necessary skills and knowledge to perform their work safely and effectively.

In short, accreditation is a formal recognition of an institution or program's quality, certification is the process of demonstrating that an individual or organization has met certain criteria or standards, and licensure is the process of granting permission to engage in certain activities.

### Tip 2 - Quality assurance versus continuous quality improvement

Quality assurance and continuous quality improvement ensure that institutions, programs, or services meet defined standards of quality and effectiveness.

Quality assurance is an evaluation process that assembles the information necessary to ensure that an institution, program, or service meets established standards. It typically involves self-study, peer review, and a site visit by an independent agency. Once the institution, program, or service has been deemed to meet the established standards, it may be accredited or otherwise recognized as being of high quality.

Continuous quality improvement focuses on ongoing evaluation and improvement of an institution, program, or service. It involves regularly assessing the institution, program, or service against established standards and identifying areas for improvement. Continuous improvement also involves implementing changes and improvements that enhance the quality and effectiveness of the institution, program, or service over time.

In summary, quality assurance ensures that institutions meet established standards, while continuous improvement is an ongoing process of evaluation and improvement. Both processes are important in ensuring that institutions, programs, or services are of high quality and effective in achieving their goals and objectives.

### Tip 3 - Process Outputs versus process outcomes

Outputs and outcomes are related but distinct entities. Together, outputs and outcomes provide important information about the effectiveness and impact of a program, project, or policy.

Outputs are the products, services, or other results that are produced by a program, project, or policy. These can include the number of people served, the number of units produced, or the amount of money spent. Outputs are typically measured in quantitative terms.

Outcomes are the changes or effects on a population that are produced by a program, project, or policy. These may include deceased hospital and emergency department usage for patients with diabetes or asthma, or lower rates of obesity. Outcomes may be expressed in qualitative terms and are often used to assess the impact or value of a program, project, or policy.

In short, outputs are the results that are produced by a program, project, or policy and offer no or little difficulty to be measured and reported. Outcomes are the changes or effects that are produced by those results; can often be subjective and therefore more difficult to operationalize and report.

### Tip 4 - Assessment versus evaluation

Assessment and evaluation are two closely related concepts that are often used interchangeably, but they actually may refer to different things. In fact, in American English, the two words have slightly different connotations: ‘assessment’ is typically used to refer to the process of measuring knowledge or skill, while ‘evaluation’ is used to refer to the process of making judgments about the value or quality of something. Interestingly, such difference is less obvious in British English, in which the terms ‘assessment’ and ‘evaluation’ are often used interchangeably to refer to the process of determining the worth, value, or quality of something, such as a program or intervention. This has generated an interesting debate in the English-speaking educational communities, but interestingly its distinction (or better the lack of distinction) becomes even more complex, as in several languages there is even no distinction for these concepts as they are encapsulated in a single word (e.g., in Portuguese or Spanish the concept is within the concept of a single word: ‘avaliação’ or ‘evaluacion’, respectively).

It is also important to separate these concepts at the individual and institutional level.

At the level of the individual, assessment is the process of measuring a student's knowledge, skills, and abilities or a characteristic of an institution to determine their level of understanding or progress. This can be done through a variety of methods adequate to the purpose. Assessment provides a snapshot of a student's current abilities. Formative assessment guides teaching and learning. Summative assessment measures mastery of knowledge and skills necessary to advance in training or begin unsupervised practice. Knowledge is often measured by written and oral examinations. Professional skills, attitudes, and behaviors are more often measured by observation of performance in actual or simulated practice. ( Epstein, 2007).

Evaluation, on the other hand, is the process of interpreting and making judgments about the information gathered through assessment and by comparison with established standards. Evaluation often involves judgments about the quality or value of a student's/professional’s work and their level of mastery or proficiency.

In contrast, institutional assessment and evaluation refers to the process of measuring and evaluating the overall performance and effectiveness of an organization, such as a medical school. The institutional process of assessment and evaluation typically involves assessing and evaluating the performance and effectiveness of an institution as a whole. This might include the assessing the quality of programs, services or policies or the evaluation of the management or governance structures or measuring the overall impact of the institution on its stakeholders. This process may use quantitative or qualitative data, such as surveys, interviews, reports or observations.

In short, assessment is the process of gathering information, while evaluation is the process of interpreting that information and making judgments based on it. Together, assessment and evaluation provide important information that can help in active monitorization of progress and implementation of future processes and plans.

### Tip 5 - Competence, Competencies, and Entrustable Professional Activities

Competent health care providers habitually demonstrate the ability to integrate their knowledge, skills, and attitudes into a set of behaviors that maintain and improve the health of their patients. The “portfolios” of individual physicians, nurses, and allied health therapists contain varying sets of competencies according to an individual’s scope of practice.

Over the past two decades, educators have focused on defining the specific and observable activities that physicians, nurses, and allied health care providers are expected to demonstrate at the completion of training. These are sometimes referred to as ‘entrustable professional activities’ (EPAs) and may be as general as ‘Manage the airway in a patient requiring a general anesthetic’, or as specific as ‘insert an endotracheal tube’ depending on the context of the evaluation, the level in training of a student, and the point of view of the authors of a set of competency domains. In either case, successful demonstration of an EPA requires integration of knowledge, skill, and judgment.

### Tip 6 - Standards, rubrics, criteria and benchmarks

Standards, rubrics, criteria, and benchmarks are related concepts in accreditation and assessment.

Standards are performance levels that define minimum expectations. They may be a simple as the percentage of correct answers necessary to pass an examination, or as complex as the duration and content of the curriculum required for the award of a medical degree or the quality of an institution. Standards are generally established by groups of experts and stakeholders in professional organizations, accrediting bodies, and government agencies. 

Rubrics specify the components of an individual standard or provide a comprehensive list of the components and standards. Rubrics may provide a scoring system for evaluating performance. For example, a rubric for evaluating a college's program in accounting might include criteria such as the quality of the faculty, the curriculum, and the resources available to students, and it might provide a scoring system that ranges from ‘unsatisfactory’ to ‘exemplary’.

Criteria are specific factors or characteristics that are used to evaluate the performance or quality of something. For example, a teacher may use criteria such as accuracy, organization, and clarity to evaluate a student's essay. An accrediting agency may use the percentage of students who successfully complete a course of study or the frequency of complaints of harassment among students or faculty as indications of student well-being.

Benchmarks compare the performance or quality of something against a standard or reference point. For example, a health care system may use the comparison of average of glycosylated hemoglobin concentrations within its population to national benchmarks as a measure of the effectiveness of its outpatient and education programs.

In summary, standards are used to set the minimum acceptable level of performance or quality. Rubrics are collections of standards used as a framework for evaluation, criteria are used to evaluate performance or quality, and benchmarks are used to compare performance or quality against a standard or reference point.

### Tip 7 - Scores, grades, reliability and validity of exams

Multiple choice examinations, oral examinations, and essay exams are facts of academic life. Formative examinations measure progress and guide teaching and learning. Faculties, licensing agencies, and specialty boards use summative examinations to measure knowledge and predict future performance in actual practice. The results of examinations may be reported as pass/fail, a percent correct, a two- or three-digit standard score, or a letter grade. Regardless of the reporting format, the percentage of correct responses required to meet the minimum competency standard is of critical importance. Examinations are considered to be reliable if it is likely that the student would get a similar number of items correct on a retest of the material covered in the exam. Examinations are considered valid if they accurately reflect the content domain under consideration (face validity) or represent the desired performance outcomes of the content domain (outcomes-based validity; Cook *et al.,* 2015).

Examinations composed of single best answer multiple choice questions are usually scored on a percent correct basis. In high stakes large scale examinations, the percent correct score may be converted to a two- or three-digit standard score. In common practice, a score of 65% (+/- 5%) is often used as indication of minimum competency. This is sometimes called the ‘cut’ score. 

The interpretation of the results, essay, and oral examinations is more subjective. In some cases, scores may be based on the number of prespecified criteria met by a candidate and may reported on numerical scales. In other cases, grading may be holistic and represent a quality judgement. Typically, these are reported as letter grades ranging from F (failure) to A (excellent). A grade of C- or D usually is considered to be the minimum standard of quality and corresponds to a score of 65%. A grade of A implies a level of achievement equivalent to a score of 90% or better. 

Faculties often use the results of examinations to benchmark performance across student cohorts, or to compare outcomes with other universities. Variability in exam content, difficulty, and delivery adversely affect the reliability of such comparisons.

Briefly, scoring is the process of assigning a numerical value to an individual's performance on a test or other assessment, and grading is the process of assigning a letter or other symbolic value to indicate the level of proficiency that a student has achieved.

### Tip 8 - Quality versus excellence

Quality and excellence are both important concepts in the fields of education, health care, and other fields. However, there are some key differences between these two terms.

Quality is a quantitative term that refers to the degree to which something meets established standards or requirements. For example, a product or service may be considered of high quality if it meets the specifications and requirements set forth by its provider. It may be of low quality if it consistently fails to meet the same specifications. Quality may also refer to the level of satisfaction or value that a product or service provides to its users. The term ‘quality’ should be not used as a substitute for excellence.

Excellence, on the other hand, refers to the state of being exceptionally good or outstanding. Excellence goes beyond meeting established standards or requirements; it involves achieving a level of exceptional performance or achievement. For example, students may be considered excellent if they consistently earn high grades. A health care system may be considered excellent if it consistently exceeds its users' expectations.

In summary, quality refers to the degree to which something meets established standards or requirements, while excellence refers to the state of being exceptionally good or outstanding.

## Conclusions

Clear and consistent use of the terminology of accreditation and assessment processes ensures clarity and consistency. This, in turn, ensures that these processes are fair, transparent, and objective. Furthermore, consistency promotes understanding and collaboration among all stakeholders. By establishing a common language and set of definitions, institutions, programs, or services, accrediting bodies, and other stakeholders can more easily communicate and work together to achieve their goals.

## Data Availability

No data are associated with this article.

## References

[ref-1] Amaral E Norcini J : Quality assurance in health professions education: Role of accreditation and licensure. *Med Educ.* 2023;57(1):40–48. 10.1111/medu.14880 35851495

[ref-2] Bandiera G Frank J Scheele F : Effective accreditation in postgraduate medical education: from process to outcomes and back. *BMC Med Educ.* 2020;20(Suppl 1):307. 10.1186/s12909-020-02123-3 32981523 PMC7520979

[ref-3] Bendermacher GWG Dolmans DHJM de Grave WS : Advancing quality culture in health professions education: experiences and perspectives of educational leaders. *Adv Health Sci Educ Theory Pract.* 2021;26(2):467–487. 10.1007/s10459-020-09996-5 33047262 PMC8041707

[ref-4] Cook DA Bridges R Ginsburg S : A contemporary approach to validity arguments: A practical guide to Kane’s framework. *Med Educ.* 2015;49(6):560–75. 10.1111/medu.12678 25989405

[ref-5] Epstein RM : Assessment in medical education. *N Engl J Med.* 2007;356(4):387–96. 10.1056/NEJMra054784 17251535

[ref-6] Frank JR Taber S van Zanten M : The essential enterprise: the critical role of accreditation in the 21st century. *BMC Med Educ.* 2020;20(Suppl 1):304. 10.1186/s12909-020-02120-6 32981522 PMC7520973

[ref-7] Stalmeijer R Whittingham JRD Bendermacher GWG : Continuous enhancement of educational quality – fostering a quality culture: AMEE Guide No. 147. *Med Teach.* 2023;45(1):6–16. 10.1080/0142159X.2022.2057285 35469546

