# Supplementary material for: A hitchhikers’ guide to the terminology of accreditation processes for health professionals and institutions
Source: MedEdPublish (2016). 2023 Feb 27;13:11. [Version 1] doi: 10.12688/mep.19566.1 (PMC10652034; doi:10.12688/mep.19566.1)
Supplement: Peer review history

# Reviewer report: Reviewer response for version 1

**DOI:** 10.21956/mep.20960.r33691

**Published:** 2023-11-15

**Authors:** Swathi K S

**Affiliations:** 1 Department of Social and Health Innovation, Prasanna School of Public Health, Manipal Academy of Higher Education, Manipal, Karnataka, India

This work is useful for the new entrants to the accreditation domain.

 It describes the terms commonly used in quality improvement and accreditation context. 

 I feel the authors need to cite the original sources which they have considered for describing the various terms in this manuscript.

 It is a work that could be published and will contribute to future works in this domain.

If evidence from practice is presented, are all the underlying source data available to ensure full reproducibility?

Yes

Is the topic of the practical tips discussed accurately in the context of the current literature

Yes

Are all factual statements correct and adequately supported by citations?

No

Are the conclusions drawn balanced and justified on the basis of the presented arguments?

Yes

Are arguments sufficiently supported by evidence from the published literature and/or the authors’ practice?

Partly

Reviewer Expertise:

Healthcare Quality Management

I confirm that I have read this submission and believe that I have an appropriate level of expertise to confirm that it is of an acceptable scientific standard.

# Reviewer report: Reviewer response for version 1

**DOI:** 10.21956/mep.20960.r32981

**Published:** 2023-03-22

**Authors:** Angel Centeno

**Affiliations:** 1 Department of Biomedical Education, Faculty of Biomedical Sciences, Austral University, Buenos Aires, Argentina

The work is very useful for those who start in medical education.

 It describes several of the most frequently used terms, and in some cases, they are used without knowing them in depth.

 I think it is a contribution to the community of educators in health sciences.

 In my opinion, the title does not precisely reflect the content of the article. It refers not only to institutional accreditation, but also to other topics such as evaluation, quality, and competencies. I would try to extend or rephrase the title to consider these issues as well.

 Items related to accreditation are very clearly described.

 There is some difficulty in explaining the differences between assessment and evaluation, and these considerations are not clear. In this case, language differences, as explained in the manuscript, are central to the confusion.

 It is a work that deserves to be published and that will contribute to the development of future health sciences educators.

If evidence from practice is presented, are all the underlying source data available to ensure full reproducibility?

Yes

Is the topic of the practical tips discussed accurately in the context of the current literature

Yes

Are all factual statements correct and adequately supported by citations?

No

Are the conclusions drawn balanced and justified on the basis of the presented arguments?

Yes

Are arguments sufficiently supported by evidence from the published literature and/or the authors’ practice?

Yes

Reviewer Expertise:

Medical Education

I confirm that I have read this submission and believe that I have an appropriate level of expertise to confirm that it is of an acceptable scientific standard.
